# Thermal ablation combined with percutaneous injection of amphotericin B for the treatment of chronic pulmonary aspergillosis: a case series

**DOI:** 10.3389/fmed.2025.1557549

**Published:** 2025-04-16

**Authors:** Xiaofeng Liu, Fuyao Nan, Peng An, Kangli Deng, Lei Li, Hongwu Wang

**Affiliations:** ^1^Department of Respiratory Medicine, Dongzhimen Hospital, Beijing University of Chinese Medicine, Beijing, China; ^2^Beijing University of Chinese Medicine, Beijing, China

**Keywords:** pulmonary aspergillosis, thermal ablation, triazole drugs, amphotericin B, drug injection

## Abstract

**Objective:**

To explore the efficacy of thermal ablation combined with percutaneous injection of amphotericin B in the treatment of chronic pulmonary aspergillosis.

**Case reports:**

Case 1 was a 66-year-old female, whose computed tomography (CT) showed a space-occupying lesion in the upper lobe of the right lung. Case 2 was a 48-year-old female, and her CT scan revealed an eccentric cavity in the upper right lung accompanied by the formation of an aspergilloma. Case 3 was a 40-year-old male, and his CT indicated the formation of an aspergilloma within the cavity in the upper lobe of the left lung. All three patients had a history of pulmonary tuberculosis and were diagnosed with pulmonary aspergillosis in other hospitals. Due to the unsatisfactory effect of oral triazole drugs, they finally came to our hospital to receive the treatment of thermal ablation combined with percutaneous injection of amphotericin B. They recovered well after the operation without significant complications.

**Results:**

During the follow-up period ranging from 1 month to 2 years after discharge, the patients reported no discomfort symptoms such as hemoptysis or chest pain. CT scans showed that the lesions were stable and even absorbed compared with those before treatment. The patients recovered well.

**Conclusion:**

The combination of thermal ablation and percutaneous injection of amphotericin B in the treatment of chronic pulmonary aspergillosis can significantly improve the clinical symptoms of patients and enhance their quality of life. It may become a treatment method for chronic pulmonary aspergillosis.

## Introduction

The genus Aspergillus is the most common pathogen causing pulmonary fungal infections. Among them, *Aspergillus fumigatus* has the strongest pathogenicity and is the most common one. It is widely present in our living environment. Under normal circumstances, Aspergillus inhaled into the airway can be cleared from the lungs. However, when the body’s defense mechanisms are impaired, pulmonary aspergillosis will occur (1). Chronic pulmonary aspergillosis (CPA) is one of the forms, which mainly affects patients with pre-existing structural lung diseases and most commonly occurs secondary to tuberculosis (2, 3). Most of the time, CPA is difficult to identify because it often presents symptoms like those of other respiratory diseases, such as persistent cough, fever, chest pain, and hemoptysis, which leads to delayed diagnosis or misdiagnosis (4). The current treatment approach recommends the use of mold-active azole drugs as the first-line antifungal treatment, for patients who are contraindicated to use azole drugs, liposomal amphotericin B is the preferred alternative drug (5). However, drug resistance in drug treatment has gradually emerged. Surgical treatment is recommended for the treatment of a single aspergilloma to prevent life-threatening bleeding. Nevertheless, surgical treatment is often limited by poor respiratory function, making patients unsuitable for surgery, and it must be carefully selected after careful consideration of potential risks of postoperative complications and disease recurrence (6, 7). There are hardly any reports on alternative salvage treatments when both drug and surgical treatments for CPA have failed. Percutaneous thermal ablation has been proven to be an effective way to treat benign and malignant tumors in various tissues. However, there are few reports on the use of thermal ablation for the treatment of CPA. Meanwhile, clinical studies have shown that percutaneous injection of amphotericin B can achieve good treatment results, and the minimum inhibitory concentration for *Aspergillus fumigatus* is much lower than the intracavitary concentration of infused antifungal drugs. In this study, we reported three cases of CPA that received the treatment of thermal ablation combined with percutaneous injection of amphotericin B and finally recovered. The follow-up ranging from 1 month to 2 years after the operation showed that the combination of thermal ablation and percutaneous injection of amphotericin B had a significant effect. The symptoms of chest pain and hemoptysis in the patients disappeared, and the follow-up CT scans showed that the lesions were calcified without progression. The treatment of CPA with thermal ablation combined with percutaneous injection of amphotericin B may provide a new clinical treatment strategy for such patients.

## Case introduction

### Case 1

**Figure 1:**
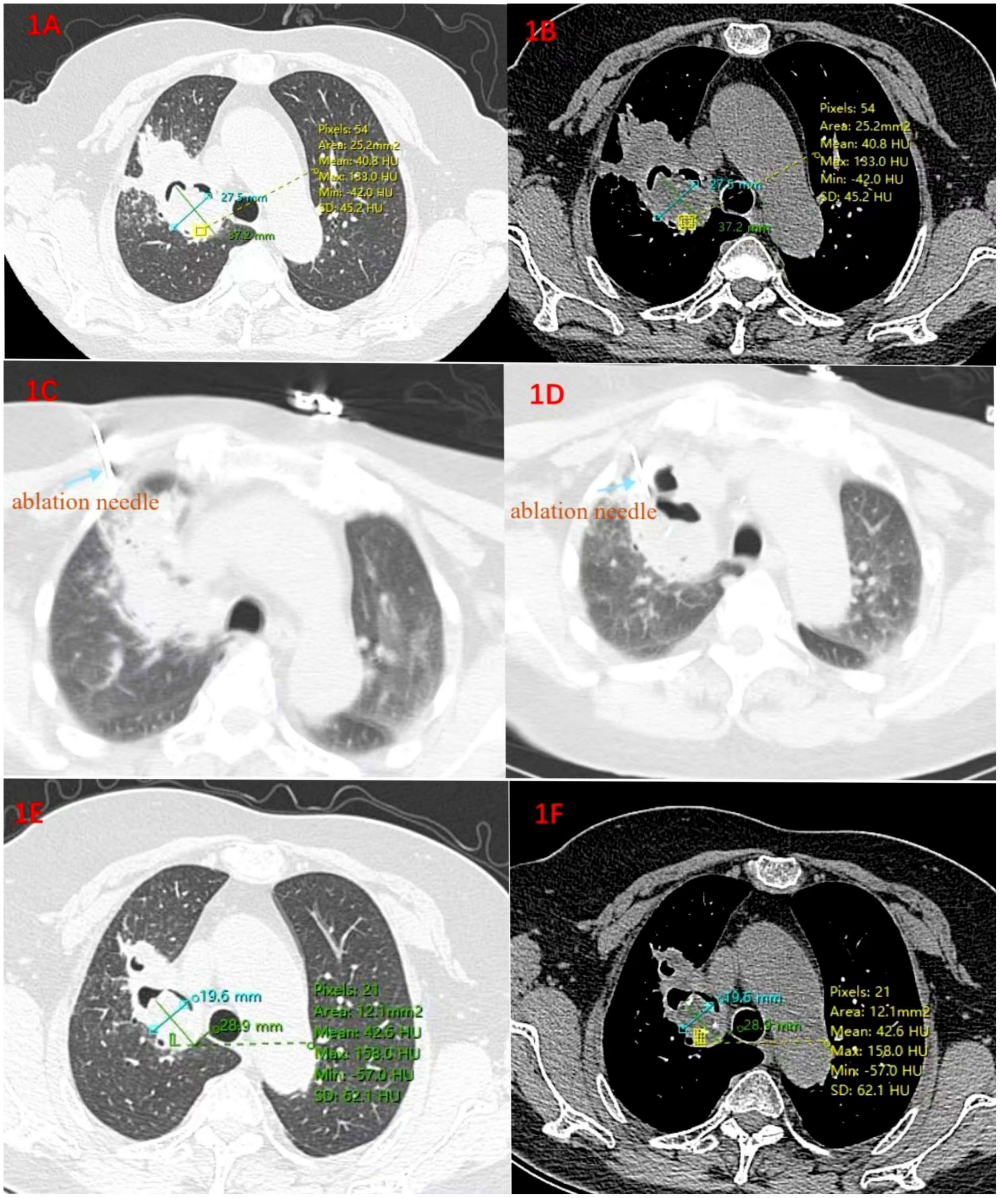
**(A)** Lung window and **(B)** Mediastinal window. On April 21, 2022, before the operation, a mixed-density mass accompanied by cavity formation was seen in the upper right lung, and fibrosis was present around the cavity; **(C,D)** The process of microwave ablation; **(E,F)** On November 17, 2022, 4 months after the ablation operation, the volume of the lesion has decreased by 45% compared with that on April 21, 2022.

A 66-year-old female with a history of pulmonary tuberculosis. In October 2020, the patient had a chest CT scan due to chest pain, which suggested a space-occupying lesion in the upper lobe of the right lung and considered the possibility of pulmonary fungal infection. Hospitalization was recommended, but the patient did not get hospitalized as she did not feel any obvious discomfort. One week ago, she began to have a cough, and expectoration accompanied by blood streaks. She was admitted to our hospital on April 19, 2022. On admission, T 36.2°C, P 72/min, R 20/min, BP 140/70 mmHg, thoracic symmetry, bilateral lung percussion voiceless, bilateral lung breath sound coarse. Upon auscultation of the lungs, no obvious dry or moist rales were detected. Bronchial exploration was performed but no lesion site was found. Later, percutaneous biopsy pathology indicated that “the mucosa and lung tissue of the upper lobe of the right lung were accompanied by fungi (considered to be Aspergillus).” She was diagnosed with chronic cavitary pulmonary aspergillosis(CCPA). Five days later, microwave ablation combined with percutaneous injection of amphotericin B was carried out. During the operation, ablation was first performed at 35 W for 5 min, and then the position was adjusted to continue ablation at 35 W for another 4 min. A follow-up CT scan showed that the ablation range covered the lesion. The ablation needle was removed, and then a mixture of 15 mg of amphotericin B for injection and 2 mL of porcine fibrin sealant was injected into the lesion along the coaxial needle. The injection was carried out smoothly, and the puncture needle was removed. The patient’s vital signs were stable, and she was returned to the ward. She was discharged after 3 days of observation when her condition remained stable. On November 14, 2022, the patient was admitted to the hospital for reexamination. The reexamination CT showed that there was a mixed-density mass in the upper right lung accompanied by cavity formation, and the lesion was absorbed compared with that on April 21, 2022. To consolidate the curative effect, percutaneous injection of amphotericin B was performed. She recovered well after the operation. During the 24-month follow-up by phone, the patient reported that she had recovered well, the symptoms of hemoptysis had disappeared, and she only coughed occasionally. A repeat CT scan was performed at the local medical institution. In accordance with the official diagnosis furnished by the local hospital, no advancement of the lesion was detected (Figure 1).

### Case 2

**Figure 2:**
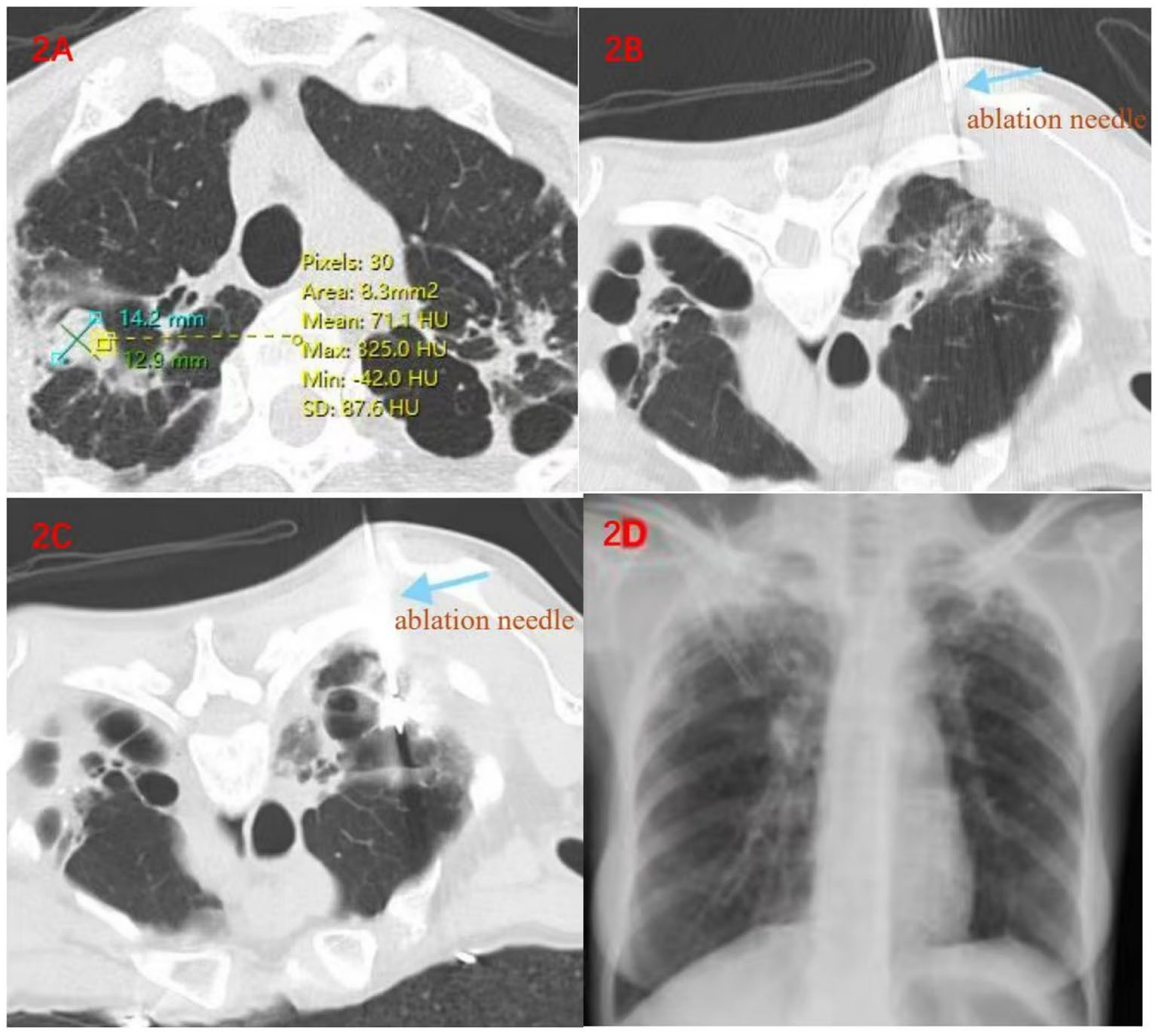
**(A)** On February 17, 2023, before the operation, a cavity was formed in the upper lobe of the right lung, and nodular soft tissue shadows and calcification foci could be seen inside it; **(B,C)**. The process of radiofrequency ablation; **(D)** A follow-up X-ray examination on the second day after the operation showed no changes such as pneumothorax or exudation, indicating that the ablation was carried out smoothly.

A 48-year-old female with a history of pulmonary tuberculosis and pelvic tuberculosis had intermittent cough, expectoration accompanied by hemoptysis for more than 7 years. Pulmonary aspergillosis had never been clearly diagnosed. During this period, she had repeated cough and expectoration accompanied by intermittent hemoptysis and had been treated with anti-infection therapy all along. Until September 2022, the patient’s cough and expectoration worsened again accompanied by hemoptysis. Fungal detection of alveolar lavage fluid was carried out in a specialized hospital in Guangzhou. Next-generation sequencing (NGS) showed that pan-Aspergillus was positive with a Ct value of 32.6. Chest CT indicated that there was an eccentric cavity in the upper right lung accompanied by the formation of an aspergilloma. She was diagnosed with CCPA. She took oral antifungal drugs for more than 5 months, but the curative effect was not good. She visited our hospital in February 2023. At admission, T 36.5°C, P 84/min, R 20/min, BP 130/80 mmHg, thoracic symmetry without abnormality, percussion of both lungs, unheard and wet and dry rales. Due to the occlusion of the lumen in the right apical segment, biopsy forceps and injection needles under the bronchoscope could not enter. One week after admission, radiofrequency ablation combined with percutaneous injection of drugs was performed. The parameters are set to power 70 W, target center temperature 80 ° C, ablate for 5 min first, and then adjust the position and continue to ablate for 5 min and 2 ablation cycles were carried out. A follow-up CT scan showed that the ablation range covered the lesion. The ablation needle was removed, and then a mixture of 15 mg of amphotericin B for injection and 2 mL of porcine fibrin sealant was injected into the lesion along the coaxial needle. The injection was carried out smoothly, and the puncture needle was removed. She was discharged without obvious hemoptysis after 3 days of observation. During the 19-month follow-up by phone, the patient reported that she had recovered well, had no hemoptysis symptoms. A repeat CT scan was performed at the local medical institution. In accordance with the official diagnosis furnished by the local hospital, no advancement of the lesion was detected (Figure 2).

### Case 3

**Figure 3:**
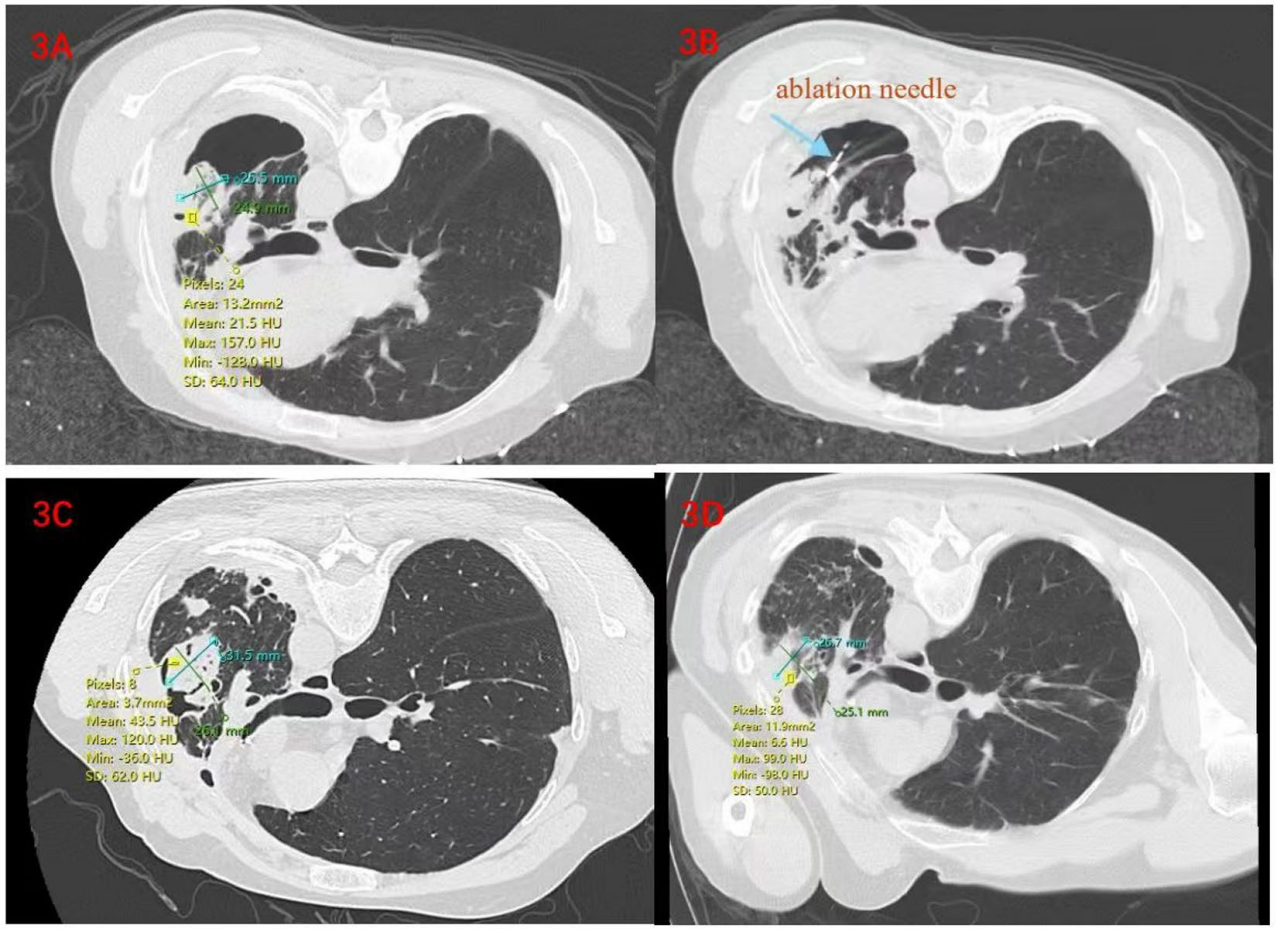
**(A)** On August 20, 2024, before the operation, changes were seen after the operation on the left lung, with an aspergilloma formed inside the cavity; **(B)** On August 26, 2024, during the microwave ablation operation; **(C)** On September 2, 2024, a follow-up examination was carried out 7 days later. Changes were observed after the ablation operation, and a parenchymal area with increased density appeared around the lesion, indicating that the ablation was complete; **(D)** On October 21, 2024, a follow-up examination was carried out 1 month after the operation, the lesion begins to absorb gradually, and the volume decreases by 18.5% compared to the previous (2024-09-02) lesion.

A 40-year-old male had intermittent hemoptysis for 13 years. Thirteen years ago, a chest CT scan due to hemoptysis showed cavitary changes in the upper lobe of the left lung. Then, a resection of the upper lobe and the dorsal segment of the lower lobe of the left lung was performed. The pathology result showed pulmonary tuberculosis combined with pulmonary aspergillosis infection. After the operation, he only took anti-tuberculosis drugs regularly for 6 months and then stopped taking the drugs as instructed by the doctor. After that, he still had intermittent hemoptysis. During this period, Bronchoalveolar Lavage Fluid (BALF) smear showed growth of *Aspergillus fumigatus*, GM test 7.63, Aspergillus IgG antibody 124.77 AU/mL, began oral voriconazole, discontinued due to adverse reactions such as vision, arterial embolization due to large amounts of hemoptysis and continued oral antifungal drug treatment, but hemoptysis symptoms still persisted. On August 19, 2024, he visited our hospital. On admission, T 37.2°C, P 100/min, R 20/min, BP 137/89 mmHg, symmetrical thorax, a 20 cm old surgical scar on the left chest wall, thick respiratory sounds in both lungs, and wet rales in the upper left lung. A chest CT scan showed that there was a cavity in the left lung accompanied by the formation of aspergilloma. Bronchoscopic exploration was carried out first, and it was found that there was no bronchus leading to the lesion. It was then decided to perform microwave ablation combined with percutaneous injection of drugs. Ablation was carried out at 35 W for 5 min. A follow-up CT scan indicated that the ablation range covered 100% of the target. Then, amphotericin B and porcine fibrin sealant were injected. After the operation, routine anti-infection and hemostatic treatments were carried out. The patient was discharged after his condition became stable. To consolidate the curative effect, he was admitted to the hospital for reexamination one month later. To further consolidate the curative effect, percutaneous puncture injection of 15 mg of amphotericin B and 2 mL of porcine fibrin sealant under the guidance of CT was carried out again. The patient recovered well after the operation, without obvious symptoms such as hemoptysis, cough or chest tightness. The follow-up CT scan showed no obvious complications such as pneumothorax or bleeding. He was discharged after 3 days of observation (Figure 3).

### Surgical technique

The patient was given oxygen inhalation through a nasal catheter, and a 250 mL of 5% glucose injection was used to open a venous access. Depending on the location of the Aspergillus, the patient was placed in a prone or supine position, and a grating was attached to the corresponding position. A plain CT scan was performed to determine the puncture point. After wearing sterile gloves, the area was disinfected routinely and then covered with a sterile fenestrated drape. The skin around the puncture point was fixed with the left hand, and a syringe was held in the right hand to perform local infiltration anesthesia with 5 mL of 2% lidocaine along the puncture point until reaching the parietal pleura. Then, the coaxial needle was held in the right hand and punctured to the set position. After removing the needle core, the ablation needle was quickly placed, and a CT scan was carried out to confirm that the ablation position was good. The ablation needle was fixed, and ablation was carried out at a certain power for a certain period of time. After adjusting the position of the ablation needle and confirming that the position was good through a CT scan, the ablation needle was fixed again, and ablation continued for a certain period of time. Subsequently, a CT scan was carried out again. When the ablation range covered the lesion, the ablation needle was removed, and a mixture of amphotericin B and porcine protein adhesive was injected into the area. After the injection of the drug, a CT scan was carried out again, and it was found that the perfusion position was good. Then, the puncture needle was removed, and pressure was applied with gauze for coverage. A CT scan was carried out again to check for pneumothorax. The patient was given oxygen inhalation and electrocardiogram monitoring and was required to lie still for 4 h after the operation.

## Discussion

CPA is a group of diseases caused by persistent pulmonary Aspergillus infection (7), with multiple manifestations. Due to the similarity between non-specific symptoms and those of other respiratory diseases, diagnosis and treatment are often delayed (8). Currently, the main treatment strategies for CPA are drug treatment and surgical treatment. Drug treatment and surgical treatment are limited due to adverse reactions and complications. There is also CT-guided percutaneous infusion of antifungal drugs for treatment, but this is only limited to some case reports (9). Intra-bronchial infusion of antifungal drugs may have curative effects, but previous studies have shown that the accuracy of drug delivery to the cavity is low in the intra-bronchial infusion method (9). Meanwhile, it is possible that the lesion cannot be found through a bronchoscope. In this study, for all three patients, the effect of triazole drugs was not significant, and adverse reactions occurred. During bronchoscopic exploration, it was found that there was no bronchus leading to the lesion, so it was impossible to carry out drug injection treatment through a bronchoscope. Therefore, the treatment plan for CPA needs to be further optimized, and it is urgent to find new treatment options.

Radiofrequency ablation is a local treatment method, which is commonly used for the treatment of malignant tumors. Through local thermal damage, energy is applied to heat the tissue to at least 60°C, inducing protein denaturation in tissue cells to obtain the maximum curative effect (10). For patients who are not suitable for surgery due to various diseases, it has become a feasible treatment option. The advantage of microwave ablation lies in its uniform heating property. It can reach a higher internal temperature of the tumor and avoid the “heat sink” effect and can generate a higher temperature in a shorter time, directly heating the tissue to a lethal temperature of 60–150°C (11–13). The genus Aspergillus is usually not heat-resistant and can be killed when exposed to a temperature of 50–60°C for 3–4 min (14). It will die when heated at 65°C for 1 min (15). Currently, only 2 patients underwent microwave ablation after being diagnosed with aspergillosis accompanied by hemoptysis. During the postoperative follow-up, scar formation was observed in the ablation area, and there was no recurrence of the disease (16). Meanwhile, clinical studies have shown that amphotericin B injection can achieve good treatment results, and the minimum inhibitory concentration for *Aspergillus fumigatus* is much lower than the intracavitary concentration of infused antifungal drugs (1, 17). Therefore, this study recorded three cases of pulmonary aspergillosis treated with thermal ablation combined with percutaneous injection of amphotericin B. After several regular treatments, the patients’ conditions have been stabilized. However, to date, there is still a lack of clinical data to guide the design of energy delivery protocols for pulmonary MWA, despite its importance. First, the ablation method is selected according to the size of the lesion. Ablation requires not only destruction of tumor tissue, but also ablation of tissue beyond the edge of the tumor by 1 cm to eliminate the lesion and prevent recurrence. Studies have shown that radiofrequency ablation (RFA) increases the risk of recurrence when the tumor is >3 cm and the ablation is incomplete (18). Case 1 had a lesion diameter of more than 3 centimeters, and case 3 had a diameter of nearly 3 centimeters, so MWA was preferred. According to the basic experimental results of Paul et al. (19), referring to previous clinical studies, and through multidisciplinary consultation in respiratory, thoracic surgery, and oncology, and based on years of clinical ablation experience, fully considering the safety of patients, it was decided to take 35 W ablation for 5 min first and then change the position for 4 min for Case 1 and Case 3 uses 35 W ablation for 5 min. The size of the lesion in Case 2 is approximately 14.2 mm by 12.9 mm, and RFA was adopted. RFA generates heat slowly, and there is a heat dissipation effect on the surrounding tissues. In order to make the temperature at the edge exceed 60°C, we set the target central temperature at 80°C. Moreover, to ensure that the ablation area covers a range of 0.5–1 cm around the lesion, we adjusted the position and carried out ablation for two cycles, with a total ablation time of 10 min. Since the power of RFA is generally between 50-150 W (20), for the sake of safety and to maintain a stable ablation effect, a power of 70 W was applied. During the postoperative follow-up, all three patients reported that the symptoms of cough and hemoptysis disappeared. The follow-up chest CT scans showed that the lesions were stable or even shrunk, and the disease had been controlled without further progression, indicating that the treatment of chronic pulmonary aspergillosis with thermal ablation combined with drug injection can achieve good treatment results, relieve the clinical symptoms of patients, and prolong the survival time of patients. Regrettably, the last follow-up was carried out by telephone. Due to the distance issue, the patient did not come to our hospital for reexamination but only had a recheck locally. We were unable to obtain the local imaging data. However, after a rigorous evaluation by the local doctor, the patient was informed that the lesion had not progressed, and this kind of curative effect assessment is meaningful.

It should be noted that cavitation in the ablation area is likely to form after ablation. It has been reported that 24–31% of patients may experience cavitation in the ablation area. The cavitation rates of radiofrequency ablation and microwave ablation are similar, but usually disappear within 2 months. The cavity is also a risk factor for the colonization of pulmonary aspergilloma and may lead to the recurrence of pulmonary aspergillosis (21–23). Therefore, we take low-power short-term treatment for pulmonary aspergillosis during ablation, and require close follow-up, regular follow-up of chest CT, observation of lesion progression, and in order to enhance the curative effect, after the end of ablation, amphotericin B is injected to avoid incomplete ablation.

## Conclusion

This case series has demonstrated the successful application of thermal ablation combined with percutaneous injection of amphotericin B in the treatment of chronic pulmonary aspergillosis. The treatment of chronic pulmonary aspergillosis with thermal ablation combined with percutaneous injection of amphotericin B is safe and effective, and patients have achieved positive results in terms of symptom relief and clinical improvement.

## Data Availability

The original contributions presented in the study are included in the article/supplementary material, further inquiries can be directed to the corresponding authors.
